# The effectiveness of microneedling technique using coconut and sesame oils on the severity of gingival inflammation and plaque accumulation: A randomized clinical trial

**DOI:** 10.1002/cre2.618

**Published:** 2022-07-20

**Authors:** Diana Mostafa, Razan Alarawi, Salma AlHowitiy, Norah AlKathiri, Razan Alhussain, Rola Almohammadi, Rawan Alhussain

**Affiliations:** ^1^ Clinical Periodontology and Oral Medicine, Faculty of Dentistry Alexandria University Alexandria Egypt; ^2^ Department of Preventive Dental Sciences Vision Colleges Riyadh Kingdom of Saudi Arabia

**Keywords:** coconut oil, gingivitis, microneedling, sesame oil

## Abstract

**Objectives:**

In our research, we evaluated the effect of coconut and sesame oils using the microneedling technique on gingival inflammation and plaque accumulation among patients with gingivitis by creating microholes in the gingiva to facilitate the concentration and entrance of the oils through gingival tissues.

**Materials and Methods:**

Twenty‐four patients with clinically diagnosed plaque‐induced gingivitis were selected from Vision dental hospital, Riyadh, KSA, and assigned to one of three groups randomly; group A consisted of eight participants who were treated with dermapen and topical coconut oil, group B had eight participants who were treated with dermapen and topical sesame oil, and group C involved eight patients who received periodontal mechanical treatment only. Postintervention gingival status and plaque status for all participants were assessed using a modified average gingival index and a plaque index at Weeks 1, 2, and 4.

**Results:**

Groups A and B experienced highly significant reductions in gingival indices, while group C showed reduced scores but was not significantly notable. On the contrary, the three studied groups exhibited no significant difference in the reduction of plaque indices when compared altogether.

**Conclusion:**

Our study demonstrated an effective novel technique that revealed a noticeable improvement in gingival status and a reduction in the average gingival index and plaque index.

## 1 INTRODUCTION

Gingivitis is a form of reversible periodontal disease, which has a high prevalence among adult populations, although it can affect all age groups (Könönen et al., 2019). However, young females commonly have a gingivitis appearance as it is aggravated by the influence of female sex hormones, which have proinflammatory effects on the oral healing process (Robo et al., 2019). Gingivitis is a multifactorial periodontal disease, the main causative factor is the local accumulation of microbial pathogens, but many factors are associated with it, such as cultural, social, occupational, and intra‐ and interindividual host factors (Kazemnejad et al., 2008).

However, plaque‐induced gingival inflammation starts when bacteria colonize on tooth surfaces, forming mature biofilms. The plaque pathogens proliferate and invade the gingival sulcus, releasing their toxins, lipopolysaccharides, and enzymes, which destroy the inner nonkeratinized epithelium and stimulate the patient's immune‐inflammatory response. This immune response starts with the recruitment of polymorphonuclear leukocytes and macrophages into the infected site with the help of complement cascades. Then, T and B lymphocytes involve the production of antibodies as well as the release of certain mediators during the process (Preethanath & Ibraheem, 2020).

Clinical characteristics of gingivitis include gingival redness, edema, change in texture, and absence of periodontal attachment loss. It is commonly painless and rarely demonstrates spontaneous bleeding, which makes most patients unaware of the disease (Trombelli et al., 2018). Nonetheless, the 2017 World Workshop on the Classification of Periodontal and Peri‐implant Diseases identified the gingivitis case by the presence of gingival inflammation at one or more sites and agreed upon bleeding on probing as the primary parameter for diagnosis of gingivitis (Chapple et al., 2018; Trombelli et al., 2018).

Gingivitis has an excellent prognosis if it is early diagnosed, regularly treated, and maintained with good oral hygiene (Trombelli et al., 2018). But if it is left untreated, more collagen loss may occur and the junctional epithelium may display apical migration and bone loss. However, the mainstay of treatment for gingivitis is debridement (mechanical anti‐infective therapy) and removal of plaque retentive factors followed by oral hygiene instructions (Peedikayil et al., 2018).

A long time ago, oil pulling (OP) was a household traditional remedy that was believed to cure many dental conditions and improve oral hygiene when practiced regularly. It has positive effects on oral health without significant side effects, such as staining and lingering aftertaste, as well as saving time and money (Shanbhag, 2016). OP generates antioxidants that damage the cell walls of microorganisms and kill them where it attracts the lipid layer of bacterial cell membranes and causes them to stick (Peedikayil et al., 2018; Sood et al., 2014). Furthermore, it decreases plaque accumulation, bleeding gingiva, malodor, dry mouth, and chapped lips. Also, it prevents dental caries, gingivitis, oral candidiasis, and periodontitis from occurring, helps to reduce tooth pain, fixes mobile teeth, and achieves vigorous oral hygiene (Ballal, 2009; Lakshmi et al., 2013).

Nevertheless, edible oils such as sesame oil, coconut oil, mustard oil, and sunflower oil were proven to remove microbes and detoxify the toxins in the laboratory in vitro studies (Hebbar et al., 2010; Thaweboon et al., 2011). Among different oils that have been used for OP practice, coconut oil is unique in its composition and is predominately composed of medium‐chain fatty acids, unlike other edible oils that are composed of long‐chain fatty acids. Also, it is composed of lauric acid, which is a saturated fatty acid that has proven anti‐inflammatory and antimicrobial effects (Peedikayil et al., 2018). In addition, sesame oil is considered to be the queen of oilseed crops because of its beneficial effects. It has antibacterial and excellent antioxidant properties that reduce lipid peroxidation, reducing free radical injury to the oral tissues (Sankar et al., 2005). Despite all these advantages, the studies on OP using coconut and sesame oils are very limited.

Furthermore, the microneedling (MN) technique is a novel Chinese puncture therapeutic modality that has been used in dermatology to enhance skin rejuvenation and improve scar texture. It is known as collagen induction therapy, which is usually combined with topical therapeutic materials (Peedikayil et al., 2018). Its technique depends on the micron‐sized needles that breach the outermost layer of the epithelium (stratum corneum), creating transient pores, to enhance the absorption of topical therapies across this layer (Vijaya Lakshmi et al., 2020). Additionally, it causes microscopic breaks in the blood vessels immediately below the epithelium, which consequently activates the natural wound healing cascade, recruiting the platelets and neutrophils to release growth factors, such as transforming growth factor (TGF)‐alpha, TGF‐beta, and platelet‐derived growth factor, promoting the deposition of collagen by fibroblasts and elastin formation, which is responsible for the tightening look (Fernandes & Signorini, 2008; Vijaya Lakshmi et al., 2020). In our study, we used a novel technique for the treatment of gingival inflammation as this is the first research to be done using the MN technique (dermapen) with topically applied natural oils on inflamed gingival tissues.

## 2 OBJECTIVE OF THE STUDY

This study aimed to assess and compare the efficacy of OP practice using coconut and sesame oils as an adjunct to MN therapy in the reduction of plaque index (PI) and gingival index (GI) in the upper sextants of gingivitis patients.

## 3 MATERIALS AND METHODS

The ethical approval was obtained by the Institutional Review Board of Vison Colleges, Riyadh, KSA with the number dent‐2020028. For every participant, informed consent was obtained before proceeding with the study. The patients were counseled before the procedure, explaining the expected outcomes and the need for multiple sessions. Additionally, consents of patients under 18 years old who particiapted in the study, were signed by their guardians.

### 3.1 Materials

**Figure 1:**
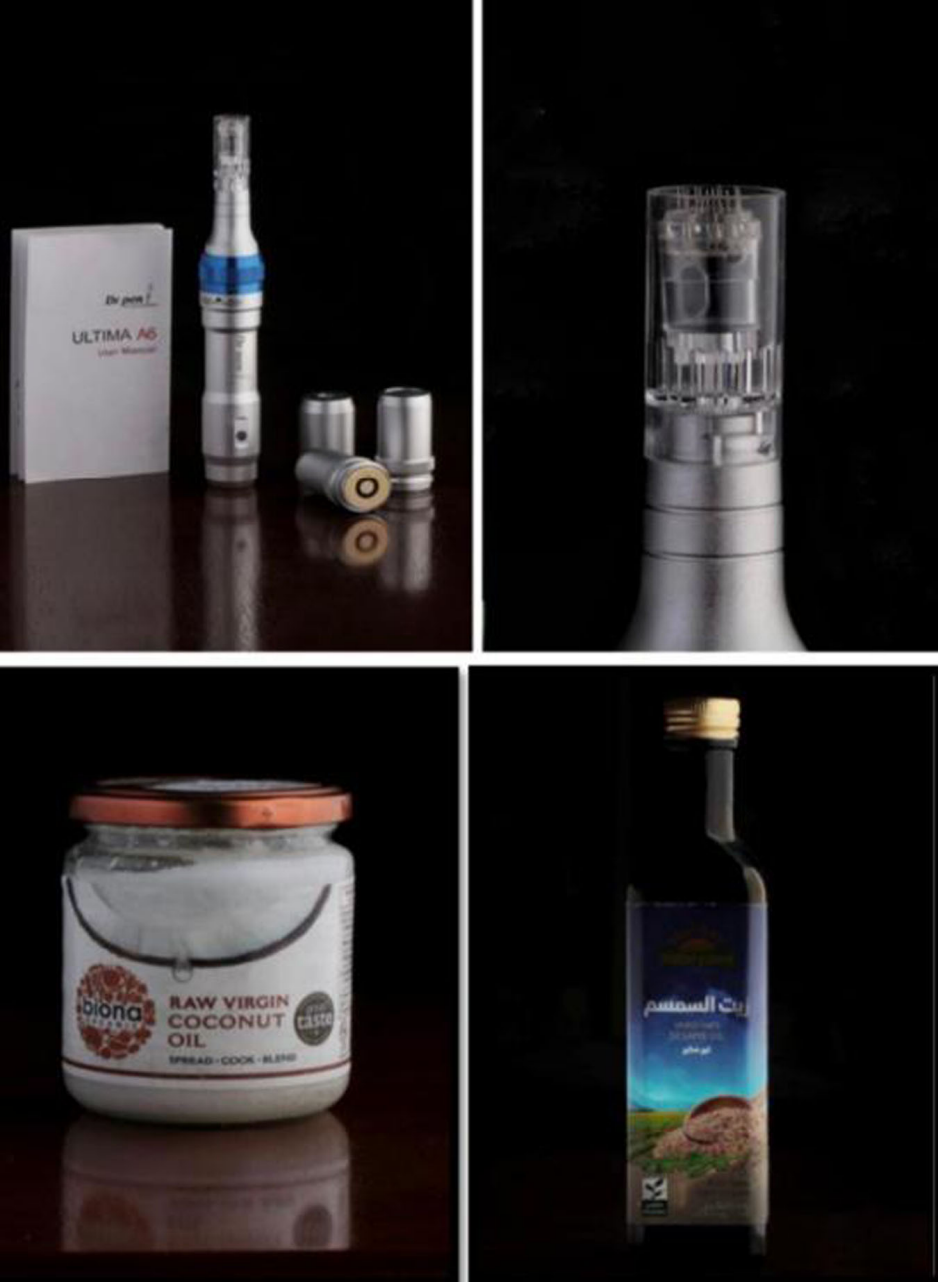
Materials used for microneedling technique, including dermapen device with reusable heads of 12 mini‐needles, raw virgin coconut oil, and unrefined cold‐pressed sesame oil

We used the dermapen device model A6, which is a pen‐like instrument with a handle, disposable heads, and guides to adjust needle length and speed (Figure 1). Each disposable head has 12 mini‐needles arranged in rows. In addition, two natural edible oils were used; 100% organic unrefined sesame oil (Natureland Company, made in Mexico), which is cold‐pressed in small batches, in a state‐of‐the‐art, low heat, oxygen‐free environment, then nitrogen flushed to maintain optimum freshness and prevent oxidation when bottled. And raw virgin coconut oil (Biona Organic Company, made in the UK), which is carefully pressed from the flesh of the fresh coconut, naturally saturated and free from trans‐fatty acids. It is not bleached, refined, or pasteurized.

### 3.2 Recruitment of subjects and study design

Our research was a selective, random interventional comparative study that included a total of 24 patients with clinically diagnosed plaque‐induced gingivitis. They were enrolled randomly between August 2020 and May 2021 from Vision Dental Hospital, Riyadh, KSA, according to the following criteria.

***Inclusion criteria***:

- Male and female health‐conscious patients.
- Age ranges from 16 to 40 years.
- Individuals suffering from moderate‐to‐severe gingival inflammation (Chapple et al., 2018).
- Redness, bleeding, and inflammation of the esthetic area (upper six anterior teeth).
- Undergone no previous treatment that had affected gingival health.
- Willingness and ability to complete the clinical trial.

***Exclusion criteria***:

- Individuals with bone loss or osteoporosis.
- Individuals with periodontitis.
- Missing one or more of the upper anterior teeth.
- Medication‐induced gingival enlargement.
- Any uncontrolled systemic disease may influence gingival health.
- Individuals with smoking and other tobacco‐related habits.
- The use of antibiotics and steroids in the previous 6 months.

### 3.3 Clinical procedures

All participants were subjected to professional mechanical scaling via a Piezoelectric ultrasonic scaler to remove calculus and soft deposits. In addition, they were given oral hygiene instructions before treatment, including proper tooth brushing techniques and flossing only. No mouthwash (MW) was prescribed to the patients. Participants were allocated randomly to either of the following groups: group A (study group) included eight participants who were treated with topical organic coconut oil using dermapen, and group B (study group) also had eight participants who were treated with topical organic sesame oil using dermapen.

**Figure 2:**
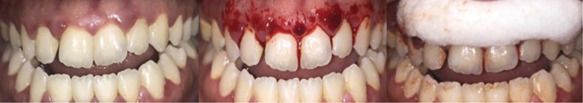
Steps of using dermapen with the application of pure organic oil

For each patient in groups A and B, local anesthesia of 2% lignocaine (1:80,000 adrenaline) was administered by infiltration technique in the anterior maxillary region. To microneedle the mucosa, dermapen needles (head/patient) were used in an intermittent stamp‐like motion on the sextant gingival area for 30–40 s/tooth with a depth of 1.5 mm. When uniform bleeding pinpoints were observed, oils were applied by cotton roll on the upper anterior region of the gingiva (gingival sulcus, gingival margin, and attached gingiva) from canine to canine in a circular motion. The cotton roll was left on the keratinized gingiva for 5 min, as shown in Figure 2. Patients were instructed to refrain from drinking acidic or hot beverages for 24 h and to not brush their teeth for 1 day to avoid any mechanical trauma to the gingiva. While in group C (control group), eight patients were advised to continue only routine oral hygiene practice in adjunct to professional mechanical scaling.

### 3.4 Clinical assessment

*Clinical examinations* included documented patient age and gender, medical and dental history, and oral care methods. Also, extra‐ and intra‐oral examinations were conducted and recorded by internship dentists. The following evaluating parameters were done by one of the investigators who were blind throughout the study period. This examiner was trained and calibrated, and an intra‐examiner variability test was performed to ensure the accuracy of the recordings.

*PI* was measured to record soft deposit accumulations on the upper six anterior teeth. Each of the four surfaces of the anterior teeth (buccal, lingual, mesial, and distal) was given a score from 0 to 3 and then areas were added and divided by four to give the accurate PI of each tooth.

- Sore 0 = No plaque.
- Score 1 = A film of plaque adhering to the free gingival margin and adjacent area of the tooth. The plaque may be seen in situ only after using the probe on the tooth surface (not seen by the naked eye).
- Score 2 = Moderate accumulation of soft deposits within the gingival pocket, or the tooth and gingival margin, which can be seen with the naked eye.
- Score 3 = Abundance of soft deposits within the gingival pocket and/or on the tooth and gingival margin that covers the interdental areas.

*GI* was calibrated to document the gingival inflammation signs of the upper six anterior teeth. Each of the four surfaces of the anterior teeth (buccal, lingual, mesial, and distal) was given a score from 0 to 3 and then areas were added and divided by four to give the accurate GI of each tooth.

- Score 0 = No signs of inflammation, bleeding, or swelling.
- Score  1= Presence of signs of mild inflammation, slight edema, and color change but no bleeding.
- Score 2 = Presence of moderate inflammation, redness, swelling, and bleeding on probing.
- Score 3 = Presence of severe inflammation, marked redness, edema, and spontaneous bleeding.

### 3.5 Postintervention comparative clinical parameters

The PI of each upper anterior tooth was calibrated, and then all final scores were added together and divided by six to calculate the average PI with the following scores and criteria:

- 0 = Excellent oral hygiene
- 0.1–1 = Good oral hygiene
- 1.1–2 = Fair oral hygiene
- 2.1–3 = Bad oral hygiene

The GI of each included tooth was measured, and then all final scores were added together and divided by six to calculate the average GI with the following scores and criteria:

- 0.1–1 = Mild gingivitis
- 1.1–2 = Moderate gingivitis
- 2.2–3 = Severe gingivitis

Then, results were tabulated according to the response of gingival status as complete improvement of gingival inflammation, reduction of GI scores, and no response to treatment or exacerbation (zero improvements) of gingival inflammation.

*Follow‐up and tissue re‐evaluation*: Each patient was reviewed for evaluation at four‐time points (four visits):

V1—Before treatment (baseline).

V2—1 week after treatment.

V3—2 weeks after treatment.

V4—4 weeks after treatment.

### 3.6 Statistical analysis

To explore the relationship between the study groups, scores and percentages were calculated. The tabulated data of each group (pre‐ and posttreatment) were statistically investigated. All data were analyzed using the analysis of variance test followed by a post hoc test for the comparison of the three study groups. Statistical analysis was performed by Package for Social Sciences software version 16.00 (SPSS Inc., Chicago, IL, USA).

## 4 RESULTS

### 4.1 Subjects' characteristics

In this clinical trial, 24 patients (16 females and 8 males) with clinically diagnosed gingivitis were divided into three equal groups, with eight patients in each group. Table 1 shows the patient characterization regarding gender and age. Each group A and group B had six females and two males, while group C included four females and four males. On comparing the three studied groups regarding gender, it was found that there was no significant difference between the two studied groups (*p* > .05). The age in group A ranged from 22 to 36 years with a mean of 27.57, and in group B, 16 to 34 with a mean of 24.29, while in group C, 27 to 40 with a mean of 29.71. There was no significant difference between the three studied groups in terms of age (*p* > .05).

**Table 1:** Characterizations of patients of the three studied groups

Characterizations of patients		Group A		Group B		Group C	
		*N*	%	*N*	%	*N*	%
Gender	Male	2	25.0	2	25.0	4	50.0
	Female	6	75.0	6	75.0	4	50.0
Age	Range	22–36		16–34		27–40	
	Mean	27.57		24.29		29.71	

### 4.2 Intragroup difference in GI

Table 2 exhibits the average GI scores at different periods of follow‐up among the three groups. At the beginning of the treatment, average GI scores were measured for all patients, where 14 patients had an average GI score of 1.2–2 (moderate gingivitis) and 10 patients had an average GI score of 2.1–3 (severe gingivitis). In group A, the average GI scores ranged from 1.2 to 3 at baseline with a mean of 2.50 ± 0.66, while after 1, 2, and 4 weeks, the GI scores were reduced to be 1.61 ± 0.67, 1.28 ± 0.79, and 1.06 ± 1.08, respectively. In group B, the average GI scores ranged from 1.3 to 3 at baseline with a mean of 2.01 ± 0.55, while after 1, 2, and 4 weeks, the GI scores decreased with *M* ± standard deviation equal to 1.41 ± 0.67, 0.98 ± 0.77 and 0.56 ± 0.44 correspondingly. While in group C, the average GI scores ranged from 1.2 to 2 at baseline with a mean of 1.67 ± 0.33, while after 1, 2, and 4 weeks, the GI scores decreased to be with *M* ± standard deviation equal to 1.21 ± 0.32, 1.27 ± 0.35, and 0.96 ± 0.76, respectively. In addition, the average GI scores after 4 weeks of treatment to the baseline among the three groups showed a highly significant difference in groups A and B, while group C displayed no significant difference from the baseline to Week 4 regarding average GI (*p* > .05).

**Table 2:** Comparison between the average GI scores at different follow‐up periods among the three groups

Group	Baseline	1 week	2 weeks	4 weeks	*p* Value
Group A					
Min	2	1	0	0	.001
Max	3	3	2.67	2.3	
Mean ± SD	2.50 ± 0.66	1.61 ± 0.67	1.28 ± 0.79	1.06 ± 1.08	
Group B					
Min	1.3	0.5	0	0	.002
Max	3	2.3	2	1.16	
Mean ± SD	2.01 ± 0.55	1.41 ± 0.67	0.98 ± 0.77	0.56 ± 0.44	
Group C					
Min	1.2	1	1	0	.376
Max	2	1.8	1.8	2	
Mean ± SD	1.67 ± 0.33	1.21 ± 0.32	1.27 ± 0.35	0.96 ± 0.76	

Abbreviations: GI, gingival index; Max, maximum; Min, minimum; *p* value, probability value; SD, standard deviation.

Statistically significant (the difference between the baseline and the final analysis within each group) at *p* ≤ .05.

Table 3 displays the comparison between the three studied groups regarding the average GI scores at different follow‐up visits. At baseline, Weeks 1 and 2, the three studied groups showed no significant difference regarding average GI (*p* > .05), while after 4 weeks, it was found that there was a highly significant decrease in GI scores between the three groups (*p* < .05).

**Table 3:** Comparison between the three studied groups regarding average GI scores

	Sum of squares	Mean squares	*p* Value
Average GI W1	2.480	1.240	.459
Average GI W2	0.560	0.280	.643
Average GI W3	0.406	0.203	.448
Average GI W4	0.950	0.475	.028

Abbreviations: GI, gingival index; W, week.

Statistically significant at *p* ≤ .05.

### 4.3 Intragroup differences in PI

Table 4 demonstrates the average PI scores at different periods of follow‐up among the three groups. In group A, the average PI scores ranged from 1.3 to 2 at baseline, with a mean of 1.62 ± 0.32. After 1 week, the average PI scores ranged from 0.3 to 1.7 with a mean of 0.93 ± 0.51. The change was not significant. Also, after 2 weeks, it was found that there was no significant change in the PI scores as they were 0.66 ± 0.39. After 4 weeks, the mean PI scores decreased to be 0.48 ± 0.50. In addition, in group B, it was found that the average PI scores ranged from 1 to 3 at baseline with a mean of 1.64 ± 0.80. After 1 week, the average PI scores ranged from 0.2 to 2, with a mean of 0.98 ± 0.53. Besides, after 2 weeks, the mean PI scores decreased to be 0.78 ± 0.56. After 4 weeks, the mean PI scores decreased to be 0.48 ± 0.51. Furthermore, in group C, it was found that the average PI scores ranged from 1 to 3 at baseline with a mean of 1.52 ± 0.75. After 1 week, the average PI scores ranged from 0.2 to 2.5 with a mean of 1.37 ± 0.71. After 2 weeks, the mean of PI scores decreased to be 1.41 ± 0.76. In the 4th week, the mean of PI scores decreased to 1.34 ± 1.23. However, on comparison between the results of PI scores in the 4th week of treatment and the baseline among the three groups, all groups showed no significant difference regarding the average PI scores (*p* > .05).

**Table 4:** Comparison between average PI scores at different periods of follow‐up among the three groups

Group	Baseline	1 week	2 weeks	4 weeks	*p* Value
Group A					
Min	1.3	0.3	0.3	0	.000
Max	2	1.7	1.5	1	
Mean ± SD	1.62 ± 0.32	0.93 ± 0.51	0.66 ± 0.39	0.48 ± 0.50	
Group B					
Min	1	0.2	0	0	.217
Max	3	2	1.27	1.2	
Mean ± SD	1.64 ± 0.80	0.98 ± 0.53	0.78 ± 0.56	0.48 ± 0.51	
Group C					
Min	1	0.2	0.5	0	.979
Max	3	2.5	2.7	3	
Mean ± SD	1.52 ± 0.75	1.37 ± 0.71	1.41 ± 0.76	1.34 ± 1.23	

Abbreviations: Max, maximum; Min, minimum; *p* value, probability value; PI, plaque index; SD, standard deviation.

Statistically significant (the difference between the baseline and the final analysis within each group) at *p* ≤ .05.

Table 5 shows the comparison between the three studied groups regarding the average PI scores at different periods of follow‐up. At baseline, Weeks 1, 2, and 4, the three studied groups showed no significant difference regarding average PI (*p* > .05).

**Table 5:** Comparison between the three studied groups regarding PI scores

	Sum of squares	Mean square	*p* Value
Average PI W1	0.052	0.026	.942
Average PI W2	0.828	0.414	.328
Average PI W3	2.318	1.159	.057
Average PI W4	3.248	1.624	.139

Abbreviations: GI, gingival index; W, week.

Statistically significant at *p* ≤ .05.

### 4.4 Differences in gingival status improvement within groups

**Graph 1:**
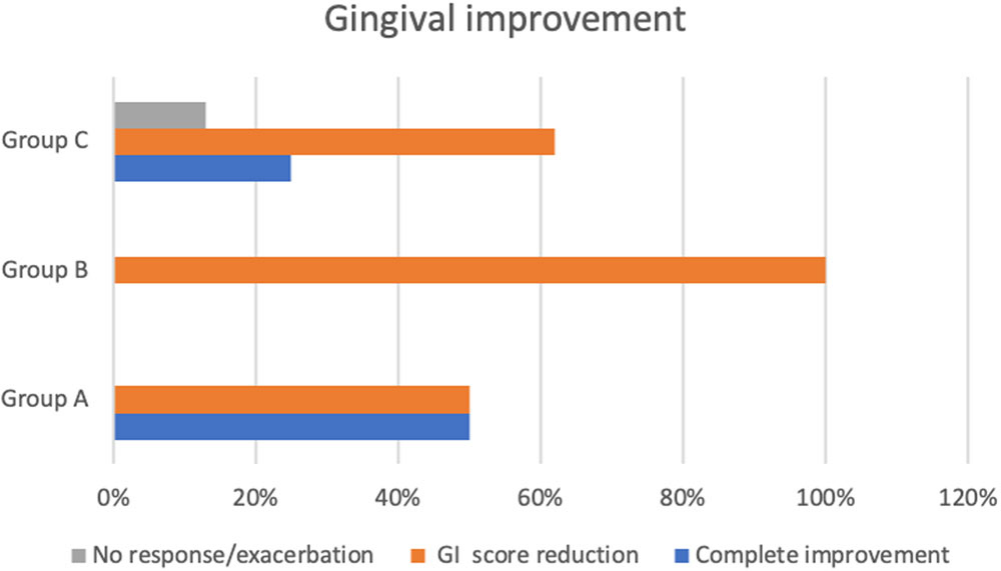
The gingival status improvements in the three groups after 4 weeks

Graph 1, 3 demonstrates the gingival status improvement between the three groups after 4 weeks of treatment, where 6 (25%) patients out of 24 patients experienced complete healing of gingival inflammation and 17 (71%) patients achieved good‐fair improvements in gingival status, while only one patient (4%) had no improvement. In group A, four (50%) patients showed complete healing and excellent improvement, and four (50%) patients displayed a reduction in gingival inflammation and good–fair improvements. While in group B, all patients (100%) showed a reduction in gingival inflammation with good–fair improvements. On the contrary, group C had two (25%) patients who showed complete healing of gingiva with excellent improvement, five (62%) patients displayed a decrease in gingival inflammation with good‐fair improvements and one (13%) patient experienced a refractory gingival inflammation and no improvement.

## 5 DISCUSSION

In our study, all participants of the three study groups received mechanical anti‐infective therapy with ultrasonic scaling, and all patients were instructed on the proper oral hygiene techniques before any treatment. In addition, we used a dermapen to introduce natural oils inside the gingival mucosa for groups A and B. Dermapen has the advantage of being wireless and reusable for different patients, as its head is disposable with mini‐needles. It is safe, more convenient, and less painful to treat small areas in the mouth, in contrast to the derma‐roller, which is a cylindrical roller with fixed‐length MNs that is difficult to be used inside the mouth. However, we used dermapen in stamp‐like motion in the anterior gingival region, but could not use it for the posterior areas due to the straight design of the dermapen's head. However, the procedure was well‐tolerated by the patients and there were no posttreatment sequelae except for slight erythema lasting for 1 day.

Furthermore, we used natural OP as a replacement for chemical MW. Chronic use of MW containing phenols and stannous fluoride produces stains and MW containing stannous and zinc salts has organoleptic problems (Amith et al., 2007; Peedikayil et al., 2018). Also, MW may cause an allergic reaction in a few individuals and a loss of taste sensation (Pemberton & Gibson, 2012). On the contrary, coconut and sesame oils are easily available, economical, and have shown numerous oral health benefits with minimal side effects (Shanbhag, 2016).

**Figure 3:**
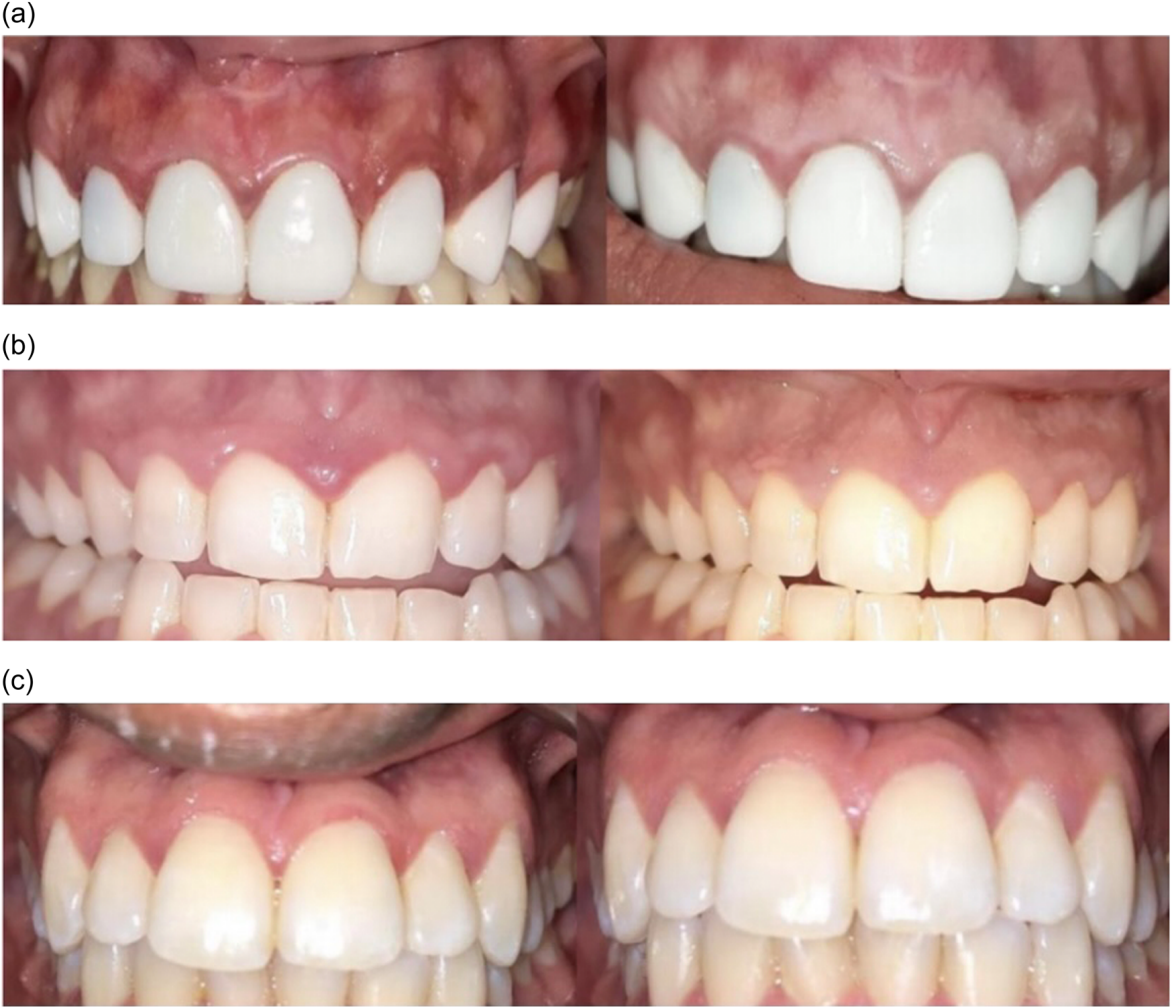
(a–c) Preoperative and postoperative pictures of the studied groups

To date, no previous research has been conducted in the evaluation of the effect of OP using MN on the severity of gingival inflammation. Accordingly, our achieved outcomes cannot be compared to any previous study. Our outcomes regarding average GI presented a statistically significant reduction on average GI from baseline to 4 weeks in groups A and B, as shown in Figure 1, 3, while group C displayed a reduction in scores but not statistically significant compared to baseline scores (Figure 1, 3). The reduction of gingival inflammation in groups A and B may be attributed to the oil‐pulling antioxidant effect, which activates the salivary enzymes and draws the toxins out of the blood (Hebbar et al., 2010; Singh & Purohit, 2011). In addition, the alkalizes in the saliva can react with the oil, leading to saponification, reducing the adhesion of plaque and gingival inflammation (Singh & Purohit, 2011).

Considering the average PI, our results revealed a reduction in PI scores comparing the baseline scores to the 4th week scores, but there was no statistically significant difference between the three groups. The reduction in PI scores in group A may be due to the reaction between the lauric acid in coconut oil and sodium hydroxide in saliva, which is responsible for the cleansing action and reduction of plaque accumulation (Asokan et al., 2009). These outcomes were in line with Peedikayil et al. (2018) who concluded that there was a statistically significant decrease in the scores of plaque and GIs when using coconut oil.

The reduction in PI scores in group B may be related to the findings of Shanmugam et al. (2001) who conducted that sesame oil has antibacterial activity against *Streptococcus mutans* and other microorganisms that contribute to plaque adhesion and bacterial coaggregation. Other studies (Asokan et al., 2009; Saravanan et al., 2013) also concluded that sesame oil reduced the severity of plaque‐induced gingivitis.

In addition, Thaweboon et al. (2011) found that coconut oil has antimicrobial activity against both *S. mutans* and *Candida albicans*, whereas sesame oil showed activity against *S. mutans* only. This confirms the antibacterial efficacy of both oils. These results were also reported by Singla et al. (2014) who concluded that there was a significant reduction in plaque scores in all chlorhexidine gel, coconut oil, sesame oil, and olive oil groups, but no significant difference was found between the four groups. While the decrease of PI scores in group C was only related to the mechanical removal of plaque and calculus, which are the main local factors that cause gingival inflammation.

Concerning the improvement of gingival inflammation, the total improvement of groups A and B was 100%, while group C was 87%. The clinical results revealed that the superiority of clinical gingival improvement was found in group A, then group B, over group C (Figure 1, 3). These outcomes were in agreement with Kaliamoorthy et al., (2018) who conducted a comparative clinical study on the topical application of coconut and sesame oils on plaque‐induced gingivitis and concluded that the maximum reduction in gingival inflammation was observed in coconut OP practice, followed by sesame oil and control group, respectively.

In addition, gingival improvement may be related to both the regenerative effect of MN and the antioxidant of coconut and sesame oils, where the MN technique affects the healing cascade by releasing various growth factors, such as platelet‐derived growth factor, transforming growth factor‐α and ‐β, connective tissue activating protein, connective tissue growth factor, and fibroblast growth factor (Falabella & Falanga, 2001). Besides, neovascularization and neocollagenesis are introduced by the migration and proliferation of fibroblasts and the laying down of the intercellular matrix, which gives the tightening look of the mucosa (Fabbrocini et al., 2009; Majid et al., 2014). Furthermore, Mostafa and Alotaibi (2022) reported that MN with ascorbic acid was effective in gingival depigmentation, resulting in the healthy pink apperance of the gingiva.

The sample number was small because of COVID‐19 circumstances, as patients visit dental clinics for emergency services only and not for esthetic reasons, which made their compliance a challenge to complete the study. Finally, one of the most positive outcomes was that the treatment with MN was well tolerated and time‐consuming; all patients in groups A and B were highly satisfied with the clinical results and had the intention of having more clinical sessions. However, the study samples reflected the findings in these selected groups of patients only. More studies with larger samples and histological analyses are necessary to confirm these results.

## 6 CONCLUSIONS

Our current study demonstrated an effective novel technique that revealed a noticeable improvement in gingival status and a reduction of average GI and PI in comparison to mechanical debridement alone. Coconut coil using MN has a superior effect on gingiva over sesame oil. Undoubtedly, coconut OP therapy using MN could be promoted as a treatment modality for periodontal diseases, especially refractory ones.

## AUTHOR CONTRIBUTIONS

All authors made substantial contributions to conception and design, acquisition of data, or analysis and interpretation of data; took part in drafting the article or revising it critically for important intellectual content; agreed for submission to the current journal; gave final approval of the version to be published; agreed to be accountable for all aspects of the work.

## FUNDING

This study was self‐funded.

## CONFLICT OF INTEREST

The authors declare no conflict of interest.

## Data Availability

Any additional data used to support the findings of this study are available from the corresponding author upon request.
